# Longitudinal Assessment of Labor Market Earnings Among Patients Diagnosed With Cancer in Canada

**DOI:** 10.1001/jamanetworkopen.2022.45717

**Published:** 2022-12-22

**Authors:** Young Jung, Christopher Longo, Emile Tompa

**Affiliations:** 1 Department of Health Research Methods, Evidence, and Impact, McMaster University, Hamilton, Ontario, Canada; 2 Klick Health, Toronto, Ontario, Canada; 3 DeGroote School of Business, McMaster University, Hamilton, Ontario, Canada; 4 Department of Economic, McMaster University, Hamilton, Ontario, Canada; 5 Institute for Work and Health, Toronto, Ontario, Canada

## Abstract

**Importance:**

To our knowledge, there have been no studies that estimated the short-, mid-, and long-term effects on cancer survivors’ labor market earnings using administrative data.

**Objective:**

To estimate the change in labor market earnings due to cancer diagnosis stratified by cancer type and age category.

**Design, Setting, and Participants:**

This population-based cohort study used a retrospective analysis of Statistics Canada’s administrative linkage file, which includes microdata from the 1991 Census, the Canadian Cancer Registry, mortality records, and personal income tax files. Participants included patients newly diagnosed with cancer from 1992 to 2008. All statistical analyses were finished on September 30, 2020.

**Exposures:**

Cancer diagnosis using the *International Classification of Diseases, Ninth Revision*, and the *International Classification of Diseases, Tenth Revision.*

**Main Outcomes and Measures:**

Annual and percent change in labor market earnings. The empirical strategy used a combination of the Mahalanobis distance and propensity score matching method and the difference-in-difference regression method to select a control group similar to the cancer survivors in this study and assess the association of the cancer diagnosis with labor market earnings, respectively.

**Results:**

A total of 59 532 patients with cancer and 243 446 patients without cancer were included in the main analysis. The mean (SD) age was similar between the matched treatment and control cohort (49.70 [8.1] years vs 49.68 [7.2] years), as was the proportion of females (0.49 vs 0.49), and the individual reported income ($37 937 [$18 645] vs $37 396 [$16 876]). The results showed the negative associations of cancer with labor market earnings. Additionally, the severity of the cancer was associated with labor market earnings, where cancer survivors with a severe type of cancer in terms of the 5-year survival rate are shown to have a larger and more persistent earnings difference compared with the control group.

**Conclusions and Relevance:**

The findings of this cohort study suggest that labor market earnings losses are associated with a cancer diagnosis. A better understanding of the loss of labor market earnings following cancer diagnosis and by cancer type can play an important role in starting a dialogue in future policy initiatives to mitigate the financial burden faced by cancer survivors.

## Key Points

**Question:**

Is cancer diagnosis associated with annual labor market earnings of cancer survivors?

**Findings:**

In this cohort study of 59 532 individuals with cancer and 243 446 without cancer in Canada, the loss of labor market earnings over time followed a U-shaped trajectory, where the lowest point or the largest labor market earnings loss occurred 1 year after the cancer diagnosis.

**Meaning:**

These findings suggest that a cancer diagnosis has the potential to change labor supply and labor market earnings in addition to the psychological costs of dealing with a cancer diagnosis.

## Teaser

This cohort study assesses changes in labor market earnings associated with cancer diagnosis stratified by cancer type and age among Canadians diagnosed with cancer from 1992 to 2008.

## Introduction

With recent advancements in medical technologies and early screening, death from all cancers combined has declined 32% since reaching its peak in 1991, and the 5-year survival rate for all cancer types combined has continued to increase from 39% in the early 1960s to 68% in 2020, indicating that more cancer survivors live longer today after a cancer diagnosis than in the past.^1,2,3^ With increasing cancer survivorship, the focus of cancer research has shifted from clinical settings to better understanding how cancer diagnosis and treatment affect families, communities, and labor. A focus has been given to the short-, medium-, and long-term labor market outcomes and related earning^1^ trajectories of cancer survivors. A substantial body of literature has documented the change in labor market outcomes due to cancer.^4,5,6^ However, most of the literature has focused on breast and prostate cancer, partly because they are the most common cancer types and have higher survival rates than other cancer types.

In this cohort study, our main objective is to estimate the association of cancer diagnosis with the yearly labor market earnings of cancer survivors from the year of diagnosis. We combine the matching technique with difference-in-differences regression analysis by using a panel of longitudinal data of Canadian individuals with and without cancer. We also objectively identify cancer types using the *International Statistical Classification of Diseases and Related Health Problems, Tenth Revision *(*ICD-10*), thus making the results comparable with other countries. Furthermore, we investigate the association between the age group and its impact on labor market earnings in the event of a cancer diagnosis.

## Methods

### Data Source

This study uses a longitudinal data set known as the 1991 Canadian Longform Census Health and Environment Cohort (CanCHEC). It is a unique data set that combines data from 5 sources: Canada’s 1991 Census of Population, the Canadian Mortality Database (CMDB), the Canadian Cancer Database (CCDB), Canadian Cancer Registry (CCR), and the T1 Family File (T1FF). The final CanCHEC data set includes approximately 2.5 million Canadian individuals who were older than 25 years in 1991.^2^ The study protocol was approved by the institutional review boards at University of Toronto and Statistics Canada. Informed consent was waived because the database used in this study contains only deidentified individual records. This study followed the Strengthening the Reporting of Observational Studies in Epidemiology (STROBE) reporting guideline.

### Sample Selection

Because the primary objective of the study is to estimate the association of new cancer diagnoses with labor market earnings, it is imperative that only cases of new cancer diagnosis were selected for inclusion. To do so, we checked the historical cancer records going back 5 years from the year of the cancer diagnosis to ensure that individuals had no prior cancer history during that time. This process was repeated for all cancer diagnoses starting 1992 to 2008. Cancer diagnosis and type of cancer were identified using the *ICD-9* and *ICD-10* in the Canadian Cancer Registry (CCR). To focus on cancer type in various subanalyses, we selected 12 different types of cancers based on the most representative cancer types in Canada. We stratified our analyses as follows: cases with any type of cancer, which includes 23 different cancer types identified in our study, the 4 major cancer types, and the 12 cancer types.^3^ Lung, colorectal, breast, and prostate cancers were included in the 4 major types. The 12 cancer types were breast, bladder, blood, cervix, colorectal, esophagus, kidney, lung, pancreas, prostate, skin, and thyroid.

Once individuals who were newly diagnosed with cancer were identified, only those individuals who showed wage-earning activities in the labor market were included in our study cohort.^7^ Earnings greater than $0 (ie, income across all paid jobs, other employment income, including tips, gratuities, and director’s fee and net self-employment income) were used as an indication for employment-related activities in the 2 years prior (ie, baseline period) to their cancer diagnosis. We included income across all paid jobs and other employment income, including tips, gratuities, director’s fee, and net self-employment income.

 After a 5-year remission period, individuals with cancer may be considered cancer-free^11,12^; thus, a follow-up period of 5 years without cancer was used to identify someone as cancer-free.^8,9,10^ We excluded anyone who had a recurrence of cancer or died during the follow-up period, as well as any individuals younger than 25 years or older than 65 years at the time of cancer diagnosis, given Statistics Canada defines active working age as 25 to 64 years.^13^ After exclusions, 59 532 individuals aged 25 to 64 years were included in our sample (30 956 men and 28 576 women).^3^Among individuals of active working age, the active group was defined as individuals aged 25 to 54 years, and the less-active group was defined as individuals ages 55 to 64 years.

### Statistical Analysis

Due to the nonexperimental nature of the population, individuals with cancer (ie, the treatment group) were likely to have different characteristics from individuals without cancer (ie, the control group). To adjust for differences in baseline characteristics, the treatment group was matched with the control group by applying the Mahalanobis distance and propensity score matching algorithm.^14,15^

The propensity score was a conditional probability of treatment assignment on observed baseline characteristics. The propensity score was generated using probability estimates from a logistic regression model in which the diagnosis of cancer (yes or no) was the binary dependent variable. Baseline characteristics were assessed as factors associated with being diagnosed with cancer. The method allows us to achieve an optimal balance of observable characteristics between the control and the treatment groups. More specifically, propensity scores were estimated using the following characteristics: disability status, household size, highest educational attainment, sex, immigrant status, and 2 prior years of labor market earnings. For baseline characteristics, such as age and sex, exact matching was undertaken. After the propensity score was generated, we applied Mahalanobis distance matching to control for any correlation between variables by which different patterns can be identified and analyzed. The caliper range was used to match cancer survivors with candidate controls based on the results of this logistic model, a range of 0.25 of the SD of the individual logit values. To ensure that the propensity score and the subsequent match were done correctly, we verified the results by estimating the propensity scores and executing the matching manually as an additional measure of quality control.

Once the treatment and the control groups were matched, we proceeded to estimate the association of cancer diagnoses with annual labor market earnings using the difference-in-difference estimator. In the statistical model, t is the year of cancer diagnosis in the treatment group and the matched year in the control group. Calendar years before and after the year of cancer diagnosis (or years matched to them in the comparison sample) are t − j and t + j, where j = 1,..., j. Labor market outcomes at t + 1, t + 2, and t + 3 are modeled as a function of a cancer indicator and individual and work characteristics. The specification is as follows:

Labor  market outcome = f × (cancer,    individual  characteristics,  work  characteristics) + ε.

Individual characteristics include age, age squared, marital status (couple or single), an indicator of having a long-term disability,^4^ and province of residence. The variables used for matching—disability status, household size, highest educational attainment, sex, immigrant status, and 2 prior years of labor market earnings—were also included in the model specification. Work characteristics are indicators for having earnings greater than $0. Quintile dummies for total earnings at t−1/2 and year dummies are also included in the model. We performed 2 tests to determine whether cancer and noncancer groups already had differing trends in the lead-up to the period where the cancer diagnosis occurred. The first test was to graph the mean earnings over time in the precancer period and observe changes. The second was to add groups-specific dummies multiplying the time trend. Both tests showed that there were no underlying time trends. All analyses were performed using STATA, version 14 software (StataCorp). All statistical analyses were finished on September 30, 2020, at the Federal Research Data Centre, Ottawa.

## Results

### Cohort Characteristics

A total of 59 532 cancer survivors and 243 446 individuals in the noncancer control group were included in the main analysis. Table 1 shows the differences in the characteristics of the cancer survivors and the noncancer group before matching. The most notable difference is the mean (SD) age between the control group and the cancer survivors. Cancer survivors were older (mean [SD], 49.70 [8.1] years) compared with the noncancer group (mean [SD], 41.03 [6.8] years). The right side of Table 1 shows the baseline characteristics among the matched group identified via Mahalanobis distance and propensity score matching. Key characteristics are similar. The mean (SD) age was similar for the cancer survivor group vs the noncancer group (49.70 [8.1] years vs 49.68 [7.2] years), as was the proportion of females (0.49 vs 0.49), and the individual reported income ($37 937 [$18 645] vs $37 396 [$16 876]), as would be expected given the matching process constrained them to be so. All results are shown in 2016 constant dollars.

**Table 1.** Baseline Characteristics of Treatment vs Control Group Matched Analysis

Characteristics	Proportion			Unmatched difference, percentage points^a^	*P* value^b^
	Unmatched treatment (n = 59 532)	Unmatched control (n = 243 446)	Matched, control (n = 143 941)		
Age, mean (SD), y	49.70 (8.1)	41.03 (6.8)	49.68 (7.2)	8.67	<.001
Disability amount for self, mean (SD) ,$	86.47 (44.8)	43.36 (32.1)	90.58 (28.8)	43.11	
Individual reported income, mean (SD), $	37 937.44 (18 645.48)	33 328.01 (17 452.49)	37 396.34 (16 876.41)	5390.57	<.001
Share of personal income to total family income	0.49	0.52	0.49	−0.03	NA
Family size	2.14	2.49	2.13	−0.35	.008
Highest level of education					
No high school	0.23	0.21	0.23	0.019	<.001
High school	0.38	0.42	0.38	−0.036	<.001
Postsecondary nonuniversity	0.21	0.19	0.21	0.015	.008
University degree	0.17	0.14	0.17	0.032	<.001
Immigrant	0.22	0.27	0.22	−0.05	.04
Sex					
Female	0.48	0.46	0.48	0.02	.032
Male	0.52	0.54	0.52	−0.02	.04
Racial or ethnic minority	0.06	0.08	0.06	−0.02	.02
Self-employed	0.07	0.14	0.08	−0.07	.008
Disabled	0.03	0.05	0.027	−0.02	.03
Marital status					
Divorced	0.07	0.08	0.08	−0.009	.03
Legally married	0.77	0.75	0.76	0.02	.03
Never married	0.11	0.08	0.12	0.03	.006
Other	0.05	0.09	0.04	−0.041	NA

Abbreviation: NA, not applicable.

^a^ This column displays the difference in means (proportion) between the cancer and comparison group before matching. Precancer characteristics were used for matching.

^b^ *P* values represent the significance of *t* test.

### Difference-in-Differences Regression Results

#### All Cancer Types

In Table 2, we show the association of cancer diagnosis over time stratified by age group and sex. Reported changes in yearly labor market earnings are compared with the baseline income, set at 1 year before the cancer diagnosis. Looking at the change in yearly earnings of both sexes, we observe the U-shaped trajectory of labor market earnings during a 5-year follow-up period. For example, cancer survivors in the active age group earned $11 244 (27.7%) less than the control group 1 year after the cancer diagnosis. Starting at 2 years after the cancer diagnosis, we observe a rebound (ie, an upward slope in the U-shaped curve) in the labor market earnings, and at 5 years after the cancer diagnosis, the cancer survivors earned $6591 (15.1%) less than the control group. Looking at the differences between sexes, we find that male cancer survivors experienced higher losses in labor market earnings than their female counterparts, but we observe a similar U-shaped trajectory for both males and females.

**Table 2.** Association of Cancer Diagnosis With Annual Labor Market Earnings by Age Groups and Sex-Matched Regression Analysis^a^

Period of cancer diagnosis	Annual labor market earnings					
	All sex		Male		Female	
	<55 years (n = 32 552)	>55 years (n = 26 980)	<55 years (n = 17 927)	>55 years (n = 14 625)	<55 years (n = 15 625)	>55 years (n = 11 355)
*t* = −1	[Reference]	[Reference]	[Reference]	[Reference]	[Reference]	[Reference]
*t* = 0						
Coefficient	−6113.8	−5232.6	−7620.2	−5716.1	−5192.3	−4764.7
*P* value	<.001	.008	NA	.03	<.001	<.001
% Change	−0.1493	−0.1396	−0.1724	−0.1115	−0.1373	−0.1602
*P* value	<.001	<.001	<.001	<.001	<.001	<.001
*t* = 1						
Coefficient	−11 244.0	−10 687.1	−12 842.4	−12 337.6	−10 124.9	−7815.4
*P* value	<.001	<.001	.005	<.001	<.001	<.001
% Change	−0.2776	−0.2616	−0.2525	−0.2403	−0.2857	−0.2621
*P* value	<.001	<.001	<.001	<.001	<.001	<.001
*t* = 2						
Coefficient	−8596.1	−10 298.8	−8898.4	−12 660.5	−7528.0	−6882.8
*P* value	<.001	<.001	.009	<.001	<.001	<.001
% Change	−0.1928	−0.2343	−0.2011	−0.2456	−0.1884	−0.2226
*P* value	<.001	<.001	<.001	<.001	<.001	<.001
*t* = 3						
Coefficient	−7124.1	−9585.8	−7743.7	−11 678.6	−6891.4	−7597.4
*P* value	<.001	<.001	.007	<.001	<.001	<.001
% Change	−0.1724	−0.2330	−0.1744	−0.2273	−0.1704	−0.2416
*P* value	<.001	<.001	<.001	<.001	<.001	<.001
*t* = 4						
Coefficient	−6817.5	−7022.1	−6841.9	−6632.0	−6818.3	−7126.1
*P* value	<.001	.004	.006	.038	<.001	<.001
% Change	−0.1636	−0.1766	−0.1616	−0.1258	−0.1684	−0.2217
*P* value	<.001	<.001	<.001	<.001	<.001	<.001
*t* = 5						
Coefficient	−6591.8	−6307.0	−6647.4	−6231.6	−6258.3	−6322.9
*P* value	<.001	<.001	.005	.002	<.001	<.001
% Change	−0.1512	−0.1703	−0.1605	−0.1143	−0.1441	−0.1913
*P* value	<.001	<.001	<.001	<.001	<.001	<.001

^a^ <55 Refers to individuals aged less than 55 at the time of cancer diagnosis. ≥55 Refers to individuals aged greater than or equal to 55 at the time of cancer diagnosis.

#### Four Major Cancer Types

We report nominal changes in labor market earnings due to the 4 cancer types—breast, colorectal, lung, and prostate (Table 3). Across all 4 cancer types, we observe U-shaped earnings trajectories, where the largest loss of earnings is observed 1 year after the cancer diagnosis, followed by a recovery of earnings starting 2 years after the cancer diagnosis. The smallest loss of labor market earnings is observed for the prostate cancer survivors for both the active ($6046 [15.2%]) and the less-active age groups ($11 324 [22.1%]) at 1 year after the cancer diagnosis compared with the control group. On the other hand, the largest loss in labor market earnings was observed for the lung cancer survivors, where the lung cancer survivors earned $23 734 (53.8%) less for the active group and $14 807 (42.1%) less for the less-active group 1 year after cancer diagnosis compared with the control group.

**Table 3.** Association of 4 Major Cancer Diagnosis With Annual Labor Market Earnings by Age Groups—Matched Regression Analysis^a^

Period of cancer diagnosis	Annual labor market earnings			
	$ Change		% Change	
	<55 years (n = 20 967)	>55 years (n = 13 977)	<55 years (n = 20 967)	>55 years (n = 13 977)
1 Year before diagnosis	[Reference]	[Reference]	[Reference]	[Reference]
Year of diagnosis				
Coefficient (SE)	−6416.9 (1660.8)	−4008.8 (1856.6)	−0.1577 (0.02284)	−0.1418 (0.02079)
* P* value	<.001	.05	<.001	<.001
1 Years after diagnosis				
Coefficient (SE)	−12 207.6 (1672.6)	−8996.2 (1884.3)	−0.3141 (0.02312)	−0.2886 (0.02177)
* P* value	<.001	<.001	<.001	<.001
2 Years after diagnosis				
Coefficient (SE)	−7535.4 (1668.5)	−9819.8 (1908.4)	−0.1980 (0.02309)	−0.2925 (0.02251)
* P* value	<.001	<.001	<.001	<.001
3 Years after diagnosis				
Coefficient (SE)	−6572.4 (1680.7)	−8983.6 (1919.4)	−0.1632 (0.02341)	−0.2615 (0.02332)
* P* value	<.001	<.001	<.001	<.001
4 Years after diagnosis				
Coefficient (SE)	−6350.2 (1693.9)	−5395.0 (1931.9)	−0.1583 (0.02365)	−0.1778 (0.02439)
* P* value	<.001	.006	<.001	<.001
5 Years after diagnosis				
Coefficient (SE)	−5118.1 (1691.2)	−4919.3 (1946.5)	−0.1366 (0.02376)	−0.1636 (0.02547)
* P* value	<.001	<.001	<.001	<.001

^a^ <55 Refers to individuals aged less than 55 at the time of cancer diagnosis. ≥55 Refers to individuals aged greater than or equal to 55 at the time of cancer diagnosis.

Table 4 and Table 5 show that cancer survivors earned less compared with the control group, and the magnitude of the decrease in labor market earnings was closely aligned with the severity of the cancer type. Skin cancer survivors, the least severe type of cancer in terms of the 5-year survival rate, have the smallest change in labor market earnings; the active group earned $907.69 (2.7%) less, whereas the less-active group earned $607.60 (1.5%) less at 1 year after the cancer diagnosis compared with the control group. At 5 years after the cancer diagnosis, the active group earned $183.50 (0.38%) less, whereas the less-active group earned $293.10 (0.05%) more compared with the control group, meaning that skin cancer survivors earned higher earnings than their noncancer counterparts. Considering the most severe type of cancer, pancreatic cancer survivors earned $28 805 (57.2%) less for the active group and $39 490 (71.8%) for the less-active group 1 year after the cancer diagnosis compared with the control group. For this group, labor market earnings show a sign of recovery starting at 2 years after the cancer diagnosis but still show a persistent loss of labor market earnings even 5 years after the cancer diagnosis.

**Table 4.** The Time-Varying Association of Cancer Types (Blood, Cervix, Kidney, and Bladder) With Annual Labor Market Earnings by Age Groups—Matched Regression Analysis^a^

Period of cancer diagnosis	Annual labor market earnings, $							
	Blood		Cervix		Kidney		Bladder	
	<55 (n = 1390)	≥55 (n = 1187)	<55 (n = 922)	≥55 (n = 2168)	<55 (n = 731)	≥55 (n = 894)	<55 (n = 966)	≥55 (n = 1391)
1 year before diagnosis	[Reference]	[Reference]	[Reference]	[Reference]	[Reference]	[Reference]	[Reference]	[Reference]
Year of diagnosis								
Coefficient (SE)	−10 882.5 (1818.7)	−8600.3 (4426.1)	−3573.2 (1174.2)	−1654.0 (4904.6)	−6716.3 (1682.5)	−4510.7 (5624.4)	−3879.9 (2004.0)	−3337.6 (4395.9)
*P* value	<.001	<.001	<.001	.008	<.001	<.001	.007	<.001
% Change	−0.2207	−0.2072	−0.0989	−0.0466	−0.1635	−0.1148	−0.0938	−0.1107
1 years after diagnosis								
Coefficient (SE)	−20 296.4 (1864.7)	−17 061.3 (4713.1)	−4834.5 (1184.8)	−3223.3 (4985.5)	−7140.2 (1727.4)	−9770.0 (5944.5)	−3711.3 (2025.2)	−6599.6 (4488.4)
*P* value	<.001	<.001	.007	<.001	<.001	<.001	.04	<.001
% Change	−0.4174	−0.4143	−0.1183	−0.0923	−0.1783	−0.2391	−0.0906	−0.2323
2 years after diagnosis								
Coefficient (SE)	−16 696.9 (1926.3)	−16 596.4 (5015.4)	−4623.2 (1196.0)	−1341.1 (5055.7)	−6073.9 (1761.9)	−10 649.8 (6256.0)	−4737.3 (2043.2)	−8182.9 (4601.8)
*P* value	<.001	.009	<.001	.004	<.001	<.001	.02	<.001
% Change	−0.3625	−0.3871	−0.1030	−0.03513	−0.1412	−0.2413	−0.1186	−0.2548
3 years after diagnosis								
Coefficient (SE)	−14 635.2 (1978.8)	−15 966.1 (5238.9)	−2590.1 (1207.0)	438.88 (5117.5)	−6268.9 (1794.3)	−10 166.6 (6412.1)	−3030.0 (2082.8)	−6967.2 (4680.7)
*P* value	<.001	.05	<.001	<.001	<.001	<.001	.007	<.001
% Change	−0.2629	−0.3573	−0.0786	0.0088	−0.1467	−0.2390	−0.0743	−0.2380
4 years after diagnosis								
Coefficient (SE)	−12 488.0 (2010.1)	−10 790.5 (5383.0)	−4172.7 (1217.1)	689.2 (5137.0)	−6683.5 (1805.9)	−9225.2 (6579.7)	−4055.6 (2085.6)	−3905.8 (4740.7)
*P* value	<.001	.03	<.001	<.001	.009	<.001	.004	<.001
% Change	−0.2234	−0.2521	−0.1072	0.0042	−0.1584	−0.2230	−0.0984	−0.1335
5 years after diagnosis								
Coefficient (SE)	−10 010.9 (2032.0)	−9324.4 (5529.9)	−4096.0 (1222.1)	443.3 (5161.1)	−5926.5 (1813.4)	−80322.9 (6622.6)	−3491.3 (2090.8)	−3771.9 (4757.1)
*P* value	<.001	.002	<.001	.004	.005	<.001	<.001	<.001
% Change	−0.2151	−0.2129	−0.0988	0.0031	−0.1428	−0.2014	−0.0832	−0.1315

^a^ <55 Refers to individuals aged less than 55 at the time of cancer diagnosis. ≥55 Refers to individuals aged greater than or equal to 55 at the time of cancer diagnosis.

**Table 5.** The Time-Varying Association of Cancer Types (Skin, Esophagus, Pancreas, Thyroid) With Annual Labor Market Earnings by Age Groups—Matched Regression Analysis^a^

Period of cancer diagnosis	Annual labor market earnings, $							
	Skin		Esophagus		Pancreas		Thyroid	
	<55 (n = 1095)	≥55 (n = 1185)	<55 (n = 1014)	≥55 (n = 830)	<55 (n = 714)	≥55 (n = 476)	<55 (n = 579)	≥55 (n = 629)
1 year before diagnosis	[Reference]	[Reference]	[Reference]	[Reference]	[Reference]	[Reference]	[Reference]	[Reference]
Year of diagnosis								
Coefficient (SE)	−1527.3 (1498.4)	−1403.3 (5314.3)	−12 088.1 (1903.6)	−19 953.2 (7237.1)	−12 193.3 (2373.9)	−14 099.0 (5899.9)	−2469.0 (1516.1)	−3891.7 (7984.0)
* P* value	.05	.009	<.001	<.001	<.001	.01	<.001	<.001
% Change	−0.03520	−0.0397	−0.2745	−0.256	−0.2673	−0.2895	−0.0558	−0.091
1 year after diagnosis								
Coefficient (SE)	−907.69 (1507.0)	−395.19 (5417.7)	−25 182.7 (2116.2)	−30 830.3 (8314.4)	−28 805.6 (2862.9)	−24 910.4 (7695.6)	−2966.1 (1522.9)	−1191.4 (8038.9)
* P* value	<.001	.005	<.001	<.001	<.001	.005	<.001	<.001
% Change	−0.02740	−0.0099	−0.5304	−0.4044	−0.5724	−0.4959	−0.0571	−0.029
**2 years after diagnosis**								
Coefficient (SE)	−426.67 (1512.7)	−2701.3 (5492.6)	−22 757.8 (4042.6)	−26 253.6 (12 434.0)	−22 757.8 (4042.6)	−26 253.6 (12 434.0)	336.41 (1521.3)	−3132.8 (8121.1)
* P* value	<.001	<.001	<.001	.05	<.001	.04	<.001	.03
% Change	−0.01818	−0.0659	−0.4783	−0.3518	−0.4448	−0.5145	0.0013	−0.082
3 years after diagnosis								
Coefficient (SE)	−839.44 (1524.3)	−6107.6 (5565.7)	−20 203.0 (2761.5)	−22 122.0 (12 218.3)	−14 372.9 (4840.3)	−39 490.3 (16 835.9)	−413.47 (1527.1)	−3138.1 (8135.8)
* P* value	<.001	.004	<.001	<.001	<.001	.03	<.001	NA
% Change	−0.01881	−0.1276	−0.4624	−0.3389	−0.2943	−0.7181	−0.0096	−0.083
4 years after diagnosis								
Coefficient (SE)	−282.0 (1532.1)	−2181.0 (5621.1)	−17 100.2 (2873.3)	−12 261.4 (12 943.0)	−11 658.2 (5064.2)	−34 773.8 (17 904.6)	−580.06 (1529.7)	−5466.0 (8201.2)
* P* value	<.001	<.001	<.001	<.001	<.001	NA	.02	.003
% Change	−0.0063	−0.0383	−0.3504	−0.1719	−0.2384	−0.6330	−0.013	−0.1322
5 years after diagnosis								
Coefficient (SE)	−183.5 (1530.6)	293.1 (5680.5)	−15 757.7 (2946.0)	−11 294.9 (13 502.3)	−10 506.5 (5490.8)	−22 728.3 (18 683.4)	−839.53 (1527.5)	−2095.6 (8207.5)
* P* value	.03	<.001	<.001	<.001	<.001	NA	NA	.01
% Change	−0.0038	0.005	−0.3347	−0.1615	−0.2094	−0.4486	−0.019	−0.0658

Abbreviation: NA, not applicable.

^a^ <55 Refers to individuals aged less than 55 at the time of cancer diagnosis. ≥55 Refers to individuals aged greater than or equal to 55 at the time of cancer diagnosis.

## Discussion

Numerous studies have examined the loss of labor market earnings due to cancer diagnosis, but to our knowledge, this is the first study to compare the loss of labor market earnings among cancer survivors with a matched control group in Canada across 12 different cancer types.^16,17,18^ More importantly, we identified that the loss of labor market earnings over time followed a U-shaped trajectory, where the largest loss occurred 1 year after the cancer diagnosis. This finding is consistent across all cancer types. There may be several reasons for the observed labor market earnings losses beyond the need to take time off work for treatment, including compromised health and function, reconsideration of time use, or involuntary job displacement. There seems to be a growing consensus that active cancer treatment may explain part of the U-shaped curve because most individuals with cancer go through a total of 3 to 12 cycles of treatments which can take up to 18 months.^10^ During this intensive treatment period, individuals may withdraw from the labor force or reduce working hours.^7^ Other studies have also found the U-shaped curve, which is explained by a portion of cancer survivors returning to work and recovering a fraction of their income during the follow-up period.^19^ Of note, the loss of labor market earnings due to cancer represents only a fraction of the overall economic burdens of cancer. To paint a full picture and understand the burdens experienced by people with cancer throughout all phases of the cancer journey, out-of-pocket costs, health care costs, and loss of productivity among caregivers must also be considered.^16,18,20^

We find heterogeneous associations where individuals with more severe cancer types, based on a 5-year survival rate, experience a larger loss in labor market earnings. The association between the magnitude of labor market earnings loss and the severity of the cancer is less well understood, but previous studies found results that corroborate our findings.^7,21^ Specifically, these studies^7,21^ found that individuals with lung and brain cancer experienced a drop of 49.3% and 45.4% in labor market earnings compared to individuals without cancer. Skin cancer survivors experienced a minor difference in income loss relative to their peers: a $2000 (5%) increase in income for males and a $970 (3.8%) decrease in income for females following the diagnosis. Additionally, about 82% of female breast cancer cases are diagnosed early in their development at Stage I or II, while 45 to 55% of pancreatic cases are diagnosed at a metastatic stage, which would require more extensive treatment. The time of cancer diagnosis and the subsequent treatment patterns may explain why patients with pancreatic cancer show a steeper U-shaped curve vs patients with breast cancer. There is some evidence to support this interpretation, but there is a need for future studies to provide more insight into the association between cancer type, type of follow-up treatments, and labor outcomes across different cancer types.^22^

Labor market activity and subsequent changes in labor market earnings following diagnosis of cancer could also be associated with age at onset: the active and the less-active labor force groups. Considering a lower labor market participation of older individuals (aged 55 years or older) who are close to a standard retirement age of 65, a cancer diagnosis may encourage older individuals to exit the labor force earlier than they would have otherwise. The initial loss of labor market earnings is more pronounced for this group of individuals, and the loss might be more persistent due to early exit from the labor force. Individuals in the active age group (aged 25 to 54 years) might experience a temporary setback in labor market earnings during active treatment and thus face higher losses but recover quickly as they return to work.^23^

### Limitations

This study had limitations. Our study calls for more rigorous data collection on cancer-related information. Despite our use of the Mahalanobis distance and propensity score matching combined with the difference-in-differences method to disaggregate the association of different cancer types with yearly labor market earnings, our ability to isolate the association of cancer diagnosis was limited by the fact that we did not have data on the severity of the disease (eg, stages of cancer) within cancer type. Unobserved differences between cancer and noncancer samples may also result in different labor market outcomes for these 2 cohorts. For instance, developing cancer may be correlated with an unhealthy lifestyle unobserved in the data (eg, smoking or poor diet). This, in turn, may depend on particular unobserved individual characteristics related to personal motivation. Individuals with such characteristics would have less chance of developing cancer (eg, by refraining from smoking) and, at the same time, have a greater likelihood of earning a higher income. Even if the confounding effect of the unobserved characteristics in this example is not particularly strong, not controlling for the average difference in unobservable characteristics of cancer and comparison groups would lead to overestimating the negative association of cancer with labor market outcomes.

The employment insurance sickness benefit provides 15 weeks of coverage in Canada, and it is usually not enough to cover the length of cancer treatment for most patients with cancer.^24^ Currently, there is no out-of-hospital support that is provided at a government level for those under 65 years. Although this varies somewhat by province, this benefit may alleviate the loss of income in the early phase of the treatment, which may minimize the impact of a cancer diagnosis on income.

Hence, our results should be interpreted as an average association of different cancer types with yearly labor market earnings. Further exploration of differences in labor market earnings by stages of cancer and treatment types will be an important area of additional research.

## Conclusions

The findings of this cohort study suggest a heterogeneous association between cancer types and labor market earnings among cancer survivors and that the age of cancer diagnosis is associated with the magnitude and duration of the labor market earnings loss. These findings may have implications in the clinical settings related to supportive care programs and government policies to encourage or support cancer survivors returning to the labor force to mitigate the losses of labor market earnings associated with cancer.

## Notes

Supplement.
Data Sharing Statement.
