# Supplementary material for: Longitudinal Assessment of Labor Market Earnings Among Patients Diagnosed With Cancer in Canada
Source: JAMA Netw Open. 2022 Dec 22;5(12):e2245717. doi: 10.1001/jamanetworkopen.2022.45717 (PMC9857413; doi:10.1001/jamanetworkopen.2022.45717)
Supplement: Supplement. — Data Sharing Statement. [file jamanetwopen-e2245717-s001.pdf]

Data Sharing Statement

Jung. Longitudinal Assessment of Labor Market Earnings Among Patients Diagnosed With Cancer in Canada. *JAMA Netw Open*. Published December 22, 2022. doi:10.1001/jamanetworkopen.2022.45717

Data
Data available: No

Additional Information
Explanation for why data not available: Individual income file needs to be stored at Statistics Canada due to privacy reason
